# Krait natriuretic peptide (KNP): a non-classical NP

**DOI:** 10.1186/2050-6511-14-S1-P67

**Published:** 2013-08-29

**Authors:** Sindhuja Sridharan, R Manjunatha Kini

**Affiliations:** 1 Department of Biological Sciences, National University of Singapore, Singapore; 2 Department of Biochemistry and Molecular Biophysics, Virginia Commonwealth University, Richmond, VA-23298, USA; 3 School of Pharmacy and Medical Sciences, University of South Australia, Adelaide, SA 5000, Australia

## Background

Cardiac homeostasis is a complex phenomenon, which is maintained by the interplay of factors that up and down regulate blood pressure. Natriuretic peptides (NPs), which cause vasodilation and increased water-electrolyte excretion, are among the most vital hormonal controls of blood pressure. NPs elicit their function by binding to membrane bound guanylyl cyclase receptor. Three mammalian NPs are known, namely ANP, BNP and CNP, which are structurally similar with a 17 residue ring and a short (5-6 residues) C-terminal tail.

## Results

Here, we describe the characterization of a novel NP from krait venom (KNP). In contrast to mammalian NPs, KNP has an extensively long 38 residue C-terminal tail, which has propensity to form α-helix, unlike other elapid NPs. The ex-vivo organ bath studies showed that the ability of KNP to relax the pre-contracted aortic strip was weaker than ANP, and it does so via a different mechanism. It arbitrates vasodilation via endothelium-dependent pathways in contrast to ANP which mediates via endothelium-independent mechanisms. Putative helical segment showed an equipotent vasorelaxation in an endothelium-dependent manner, while only the ring of KNP showed similar vasorelaxation as ANP in an endothelium-independent manner. Deletion of the C-terminal helical segment abrogates KNP’s activity suggesting its definitive role in vasorelaxation. Further with different inhibitors, we have delineated the necessity for nitric oxide, prostacyclin and endothelium derived hyperpolarization factor (EDHF) for KNP mediated vasodilation.

## Conclusion

We have established that KNP mediates vasodilation by a unique mechanism that can be attributed to its C-terminal tail.

